# Management of sub-5 mm rectal carcinoids with lymph node metastases

**DOI:** 10.1093/gastro/gou073

**Published:** 2014-10-22

**Authors:** James Wei Tatt Toh, Christopher Henderson, Takako Eva Yabe, Evonne Ong, Pierre Chapuis, Les Bokey

**Affiliations:** ^1^Department of General Surgery, Liverpool Hospital, Liverpool, New South Wales (NSW), Australia,; ^2^Department of Pathology, Liverpool Hospital, Liverpool, NSW, Australia,; ^3^South West Clinical School, University of New South Wales, Sydney, NSW, Australia,; ^4^Department of Colorectal Surgery, Concord Hospital, Sydney, NSW, Australia and; ^5^Department of Colorectal Surgery, Liverpool Hospital, Liverpool, NSW, Australia

**Keywords:** rectal carcinoid, lymph node metastases, tumour size

## Abstract

Minute (<5 mm) and small (5–10 mm) rectal carcinoids discovered during colonoscopy are generally considered to be non-aggressive, and the management and surveillance of patients with this entity are usually limited. We present the case of a 61-year-old Chinese female with multiple sub-5 mm carcinoid tumours in the rectum without any computed tomography (CT) evidence of lymph node or distant metastases. She underwent an ultra-low anterior resection for a sessile rectal polyp with the histological appearance of a moderately differentiated adenocarcinoma. Seven foci of minute carcinoids in the rectum and perirectal lymph node metastastic spread from the carcinoid tumours were also discovered on histopathology. There were no lymph node metastases originating from adenocarcinoma. This case report and review of the literature suggests that minute rectal carcinoids are at risk of metastasizing and that these patients should be investigated for lymph node and distant metastatic spread with CT and somatostatin receptor scintigraphy or its equivalent, as this would influence prognosis and surgical management of these patients. Findings relating to lymphovascular invasion, perineural invasion, high Ki-67, mitotic rate, depth of tumour invasion, central ulceration, multifocal tumours and size are useful in predicting metastases and may be used in scoring tools. Size alone is not a good predictor of metastastic spread.

## Introduction

Minute and small rectal carcinoids are generally considered to be non-aggressive, with little likelihood of metastasizing. This has led to changes in current practice guidelines, with significant supporting literature demonstrating good results from the treatment of tumours smaller than 1 cm by endoscopic mucosal resection (EMR) using band ligation or transanal submucosal excision [1, 2]. There is minimal local or distal recurrence of sub-10 mm non-metastatic carcinoid tumours following complete endoscopic removal [2, 3]. This is not the case for carcinoid tumours greater than 20 mm. Approximately 15% of rectal carcinoids are associated with metastases [4].

While tumour size remains a useful guide to the management of rectal carcinoids, size alone is not a reliable predictor of metastastic spread. In a study of 1914 carcinoids (849 of which were rectal), lymph node involvement was detected in 3.7% of cases (8 of 216) of <5 mm rectal carcinoids and 13.2% (50 of 379) of cases of 5–10 mm carcinoids, while the overall rate of metastases for those smaller than 10 mm was 9.7% (58 of 595). While the overall rate of metastases in the 10–20 mm range was 27.6% (42 of 152) and that for carcinoids greater than 20 mm was 56.7% (17/30) [5], approximately 10% of <10 mm rectal carcinoids are associated with metastases: thus, size alone is not a good predictor of metastases. A case report by Tsuboi *et al.* described a sub-5 mm rectal carcinoid with multiple liver metastatic carcinoid tumours [6]. Heah *et al.* conclude that tumour size is irrelevant in predicting malignant potential of carcinoid tumours, using the example of a patient with a 1 mm rectal carcinoid with 2 out of 12 nodes being positive [7].

We report here the management of a 61-year-old female, found to have multifocal minute (<5 mm) rectal carcinoids with a low Ki-67 of only 1%, but with lymphovascular space invasion (LVI) and metastatic carcinoid in a perirectal lymph node on definitive resection.

## Case presentation

Because of a positive faecal occult blood test (FOBT), a 61-year-old Chinese female underwent a colonoscopy that showed a sessile polyp with central ulceration just distal to the rectosigmoid junction. The polyp was snared and histopathology showed features of a moderately differentiated adenocarcinoma, which was limited to the submucosa, with no evidence of lymphovascular invasion and 0.9 mm from the excision margin. There was also a 6 mm mucosal leiomyoma, sampled from the sigmoid colon, and a large submucosal lipoma in the caecum. The patient was further investigated with a computed tomography (CT) scan of the abdomen and pelvis, tumour markers and a repeat colonoscopy with tattooing of the rectal lesion.

The CT showed no evidence of metastatic disease to solid organs, nor evidence of involvement of the mesenteric lymph nodes. There were two well-defined low-density cystic liver lesions, with the larger cyst being 6 mm in segment 6. A 15 mm enhancing myometrial nodule was also seen, consistent with a uterine fibroid. Tumour markers, including alpha-fetoprotein (AFP), carbohydrate antigen 19.9 (CA19-9), carcinoembryonic antigen (CEA) and carbohydrate antigen 125 (CA125), were all negative pre-operatively.

**Figure 1.**
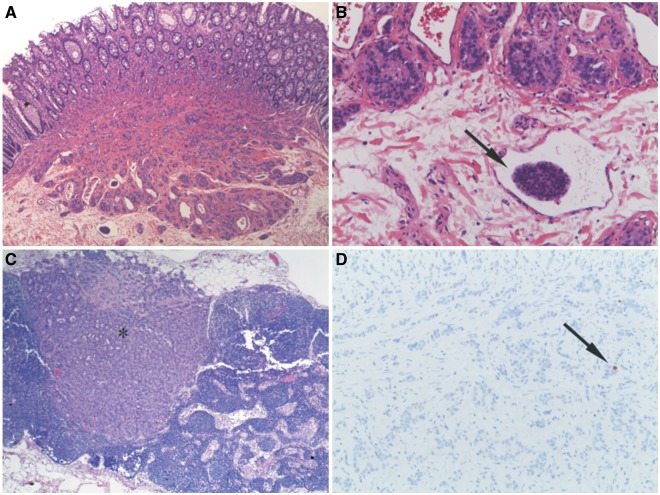
Histology of multifocal minute rectal carcinoid with lymph node metastasis. (A) Largest of the carcinoid tumours (3.5 mm) with extension into the submucosa of the rectum (magnification x20; haematoxylin & eosin). (B) Lymphovascular invasion (arrow) in the submucosa adjacent to largest tumour (magnification x100; haematoxylin & eosin). (C) Metastatic carcinoid deposit (asterisk) within pericolic lymph node (magnification x20; haematoxylin & eosin). (D) Sparse positive tumour cell nuclear staining with Ki-67 (arrow), indicating low Ki-67 index (magnification x100; immunoperoxidase method)

The patient subsequently underwent an open, ultralow, anterior resection with a defunctioning loop ileostomy. The histopathology of the resected specimen showed multifocal carcinoid tumours (7 foci) with Grade 1 features (WHO grading, 2010) ranging from 0.8–3.5 mm in size, with direct invasion into the submucosa (Figure 1A), with focal lymphovascular invasion (Figure 1B), and with perirectal lymph node involvement (Figure 1C) in one of the twenty local nodes. The Ki-67 index (Ki-67 is a nuclear protein antigen associated with cellular proliferation) was 1% (Figure 1D) and there were neither atypia nor necrosis. A single perirectal lymph node showed a 2 mm deposit of metastatic carcinoid. Immunohistochemistry confirmed that both the primary tumours and the metastasis were strongly chromogranin-positive.

## Discussion

This case suggests that the size of a carcinoid tumour alone is not a reliable indicator of metastatic disease. The largest carcinoid lesion in this patient was only 3.5 mm and yet there was one lymph node metastasis that was not detected on standard CT scan. This case study demonstrates the unpredictable nature of rectal carcinoids and suggests that it should not be assumed that minute and small rectal carcinoids do not metastasize.

### Is endoscopic management adequate for small and minute rectal carcinoids, and should additional investigations be performed to rule out lymph node metastases?

The current guidelines for rectal carcinoids are that endoscopic removal is adequate for tumours smaller than 10 mm, without central ulceration or muscularis propria invasion [8]; muscularis propria invasion can be assessed by endoscopic ultrasound. The rate of complete resections, in both EMR by band ligation and ESD, has been shown to be more than 80% [1]. However, this case report shows the importance of investigating appropriately for lymph node metastases, as even sub-centimetre rectal carcinoids metastasize. Most of the case series reported in the literature, suggesting that small and minute carcinoids do not need regular surveillance, exclude patients with lymph node metastases. Murray *et al.* followed up 18 patients with minute carcinoids without known metastases (10 out of 28 had metastases) following (i) endoscopic removal (*n = *13), (ii) transanal excision (*n = *3), (iii) transanal endoscopic microsurgery (*n = *1) and (iv) no additional invasive therapy after diagnostic endoscopy (*n = *1), with an average follow-up of 5.4 years, and found no recurrence of disease or metastases [3]. Onozato *et al.* followed up 38 patients with small carcinoids (<10 mm) without lymph node metastases, who underwent endoscopic removal for an average follow-up of 6.3 years, and found no recurrences or metastases [2]. However, when there is lymph node involvement or the lymph node status is unclear, the management algorithm changes and more aggressive investigation and surgical management are important considerations.

### Lymph node metastases from rectal carcinoids are associated with a worse prognosis and require surgical management

Lymph node metastasis, regardless of size, is associated with a worse prognosis. Lymph node involvement has been demonstrated to be associated with the development of distant metastases and significantly affects survival. In a multicentre study involving nine treatment centres in Europe and North America, 202 patients with rectal carcinoids with a median tumour size of 10 mm were prospectively identified and followed up for five and ten years [9]. Patients with lymph node metastases had a five-year survival rate of 70% and a 10-year survival of 60%. Patients with distant metastases had a four-year survival rate of only 38%. Importantly, the presence of lymph node metastases was associated with the development of distant metastases (P = 0.033) [9]. The presence of lymph node metastases associated with carcinoids is a predictor of decreased survival [10] and results in an equally poor prognosis to that of patients with lymph node metastases associated with rectal adenocarcinomas.

### The size of a carcinoid tumour is not a reliable predictor of lymph node involvement; multiple laboratory, endoscopic and radiological methods are available to assess for lymph node metastases

The size of a rectal carcinoid tumour alone is not a reliable indicator of lymph node involvement [7, 11, 12]. Various studies have proposed other methods of evaluating rectal carcinoids. A recent study, comparing the outcomes of rectal carcinoids and AJCC/TNM guidelines, showed good correlation between 5-year disease-free survival (DFS), prognosis and TNM stage. Lymph node metastases correlated best with depth of invasion (from pT1a–1.2%, T3–84.6%) [13]. Lymphovascular invasion and perineural invasion were also associated with lymph node metastases and lower survival [14]. Shinohara *et al.* recommended the use of other risk profiles, such as Ki-67 and lymphovascular invasion, as a marker for lymph node involvement [11]. Hotta *et al.* showed, in 43 patients with small rectal carcinoids, that the Ki-67 ratio was a reliable marker for predicting the metastatic potential of rectal carcinoids (sensitivity 88.9%; specificity 82.4%) [15]. The mean Ki-67 ratio in the metastatic group was 3.9% and, in the non-metastatic, group 1.0%. Cytophotometry is not commonly used but, according to Tsioulias *et al.*, the presence of diploid DNA is protective, whereas DNA aneuploidy puts the patient at higher risk of metastases [16]. Fahy *et al.* reported a Carcinoid of the Rectum Risk Stratification (CaRRS) score to accurately predict outcome, which considered size, invasion, LVI and mitotic rate [17].

Fujimoto *et al.* suggested that a CT finding of a lymph node greater than 5 mm is indicative of lymph node metastasis [10]; however, as demonstrated in this case, CT alone may not be a reliable predictor for lymph node metastases developing from carcinoid tumours. This is because carcinoid tumours are usually small, and imaging techniques with CT and MRI may not detect small or minute carcinoids or small isolated metastases [18]. Rahman *et al.* described the importance of Indium-111 octreotide single-photon-emission computed tomography (SPECT) to evaluate for lymphatic and distant metastases [19]. The use of somatostatin receptor scintigraphy is useful for identifying the presence of lymph node and distant metastases [20]. Iodine123-meta-iodobenzylguanidine (MIBG) may also be used [21].

Somatostatin receptor scintigraphy is increasingly being used pre- and post-operatively to detect small primary carcinoids and metastatic spread because it has a good sensitivity. Approximately 80–100% of carcinoids contain somatostatin receptors of five subtypes [22]. Indium-111 pentetreotide scan has an overall imaging sensitivity of 80–90% in patients with carcinoids. Somatostatin receptor scintigraphy imaging is considerably better than CT or MRI in diagnosing carcinoid tumours, particularly in the cases of small and minute carcinoids. It also allows for whole-body imaging capability. Gallium-68 labelled dotatate PET/CT scanning (also based on somastatin receptor scintigraphy) is currently being evaluated and used in the diagnosis of carcinoids. Somatostatin receptor scintigraphy with CT is becoming the initial procedure of choice for the localization and staging of carcinoid tumours.

MIBG scintigraphy may also be useful for detecting carcinoids. Many types of carcinoids synthesize and secrete catecholamines; as a result, carcinoid patients should have elevated urinary catecholamines and MIBG accumulation in carcinoid tumours and metastastic foci [23, 24]. However, the sensitivity of iodine-123 (I-123) or I-131 MIBG scintigraphy in carcinoids is only 55–70%, falling short of the 80–90% sensitivity of somatostatin receptor scintigraphy [25].

The role of endoscopic ultrasound is not clearly defined for rectal carcinoids [26] and it is not routinely used in their management. Endoscopic ultrasound is valuable for assessment of depth of tumour invasion. Kobayashi *et al.* utilized endoscopic ultrasound to assess depth of invasion and found that none of the 57 lesions that did not involve invasion of the *muscularis propria* had lymph node metastases [8]. On the other hand, invasion of the *muscularis propria* was associated with metastasis [8, 27]. The role of endoscopic ultrasound for fine-needle aspiration of perirectal nodes in the setting of carcinoid is less clear.

Both endorectal ultrasound and MRI may demonstrate perirectal infiltration, but the appearance of carcinoids under MRI is variable and difficult to interpret: they may appear hyperintense or hypointense, and some may appear only on immediate post-gadolinium images [28]. Accordingly, MRI is not commonly used for assessing carcinoid tumours.

### Multifocal carcinoids are uncommon and associated with increased risk of lymph node metastases when compared to small single carcinoids

Only a few cases of multifocal carcinoids in the rectum have been reported in the literature, including multiple rectal carcinoids in the setting of diffuse ganglioneuromatosis [29, 30] and in ulcerative colitis [31, 32]. The prevalence of lymph node metastasis has been shown to be higher (up to 22.7%) in multiple sub-10 mm rectal carcinoids than in single examples [33]. Patients with multiple rectal carcinoids may benefit from closer surveillance and more aggressive management.

### There are no consensus guidelines on post-operative management of rectal carcinoids with isolated lymph node metastases but without distant metastases

Post-operatively, carcinoids with lymph node- or distant metastases have a prognosis comparable to rectal carcinomas and should be considered as such [27]. Sauven *et al.* showed that when patients had lymph node- or distant metastases, this was associated with a poor prognosis [34]. In Sauven's cohort, all 13 patients who had lymph node involvement developed distant metastatic disease and had a median survival of only 10 months. One patient, however, survived for 9 years [34].

It has been suggested that the surveillance of carcinoid patients with isolated lymph node metastases (AJCC Stage IIIB) should include 24-hour urinary collection of 5-HIAA or fasting serum levels of 5-HIAA or serum chromogranin A levels at 3 months, as well as somatostatin receptor scintigraphy at 6 months. While Stage IV patients are offered a range of chemotherapy, somatostatin analogues and interferon-alpha, there are no consensus guidelines for management of isolated lymph node metastases without distant metastases.

Combination chemotherapies, including 5-fluorouracil [35], dacarbazine, etoposide [36] and doxorubicin [37], may prolong survival for patients with distant metastatic disease. Somatostatin analogues, such as octreotide and lanreotide, have also been shown to prolong survival. A range of ablative methods have been used for liver metastases including chemoembolization, radioembolization, radiofrequency ablation [38], hepatic arterial infusion chemotherapy [39], cryotherapy, and alcohol injection.

## Conclusion

This case demonstrates the need for accurately assessing the presence of lymph node involvement and distant metastatic spread, rather than basing management decisions solely on the size of rectal carcinoid. This is because lymph node metastases may be found even in minute carcinoids and endoscopic treatment is inadequate for rectal carcinoids with lymph node involvement. Also, confirmation of lymph node metastases will influence prognosis and future patient surveillance. The most sensitive test to localize minute and small carcinoids and lymph node and distant metastases is somatostatin receptor scintigraphy PET/CT. Post-operatively, along with urinary or serum 5-HIAA and/or chromogranin A, this is useful for surveillance. A small or minute rectal carcinoid found incidentally on colonoscopy or excision biopsy warrants further investigations to identify possible metastatic disease.
